# Comparative studies need to rely both on sound natural history data and on excellent statistical analysis

**DOI:** 10.1098/rsos.171211

**Published:** 2017-11-15

**Authors:** Dieter Lukas, Tim Clutton-Brock

**Affiliations:** Department of Zoology, University of Cambridge, Downing Street, Cambridge CB2 3EJ, UK

We certainly agree with Schradin [1] that comparative studies need to use the best information available—as well as clear definitions, up-to-date statistical techniques and common sense. We have divided our response into two sections: the first dealing with Schradin's critique of our 2017 paper and the classification of the social organization of shrews; the second dealing with three general issues that his commentary raises.

## 1. The social organization of shrews

The paper that Schradin is commenting on [2] asks whether cooperative breeders (which we define as species where non-breeding helpers assist breeders to raise their young) in mammals are more commonly found in arid environments than monogamous species (defined as species where breeding pairs of males and females remain together for more than one season) from which cooperative breeders appear to be derived [3]. It shows that, as in birds, cooperative breeders tend to live in relatively arid habitats compared to monogamous species. As we describe below, these conclusions are unaffected by differences between our classification of the social organization of shrews and the classification suggested by Schradin.

Schradin's first criticism is that the categorizations of shrews that we have used in this analysis were based on one paper on a single species. This is incorrect, for our categorization of shrews was based on more than 70 separate sources (see electronic supplementary material, appendix S1). When we published our dataset [4], we were required to reduce the associated reference list by the editors of the journal and, to comply, we removed around 75% of our 2000+ sources, often listing a single reference per species or group of species, and frequently using generic reviews for particular groups where these were available: for example, for many rodent species, we simply listed Wolff and Sherman's *Rodent Societies* [5], and for many carnivores, we listed the review by Dalerum [6]. To warn readers that our dataset was not derived from these generic references alone, we inserted a comment in the electronic supplementary material of the paper that ‘although the supplement provides a single reference for each species for further information, the classification of most species was based on information from several sources' [4]. The traditional view of social organization in shrews and the consensus of most overviews of the social organization of shrews is that breeding females are intolerant of each other and that multiple breeding females do not form coherent social groups (see [7–11]). Although the generic reference that we cited in connection with our classifications of shrews [12] contained a statement that shrews are solitary in its Introduction, it was not a general review and we agree with Schradin that it was not a suitable source to cite in this connection and that it would have been more appropriate to have listed one or more sources that provided extensive reviews or primary information, like many of those listed in electronic supplementary material, appendix S1.

Schradin's second criticism of our 2017 paper concerns differences in the classification of eight shrew species between our 2017 paper and his 2015 paper with Valomy *et al*. [13]. Three species that Valomy *et al.* classify as group living in their table (*Cryptotis parva*, *Sorex cinereus* and *Sorex ornatus*), we classify as solitary breeders (see table 1). These differences are related to contrasts in definitions. We classify species as social breeders only if multiple breeding females aggregate in coherent groups during the breeding season and classify them as solitary breeders if breeding females are intolerant of each other and do not aggregate with each other in the breeding season (the rationale for this is described in box 1). The papers that Valomy *et al.* refer to in their 2015 paper provide no evidence that female shrews are social during the breeding season and so do not allow us to classify them as social breeders. Valomy *et al.* [13] do not provide definitions of their categories but evidently used a less restrictive definition of group living. *Cryptotis parva* are reported to aggregate during the winter for thermo-regulatory reasons, but it is not clear whether females aggregate during the breeding season [22,23]; reports of sociality in *So. cinereus* describe parties of males competing to gain access to single females during the mating season but provide no evidence that multiple breeding females aggregate in coherent groups or share a common range [15]; and, while aggregations of *So. ornatus (sinuousus)* occur outside the breeding season*,* they appear to be loose and unstable and there is no indication that breeding females form coherent groups during the breeding season [14,16] (table 1).

Box 1. **Definitions of categories of social organization used in our 2013 and 2017 papers.**

The definition of the categories that we used to classify social organization in shrews and other mammals are described in the main text of our 2013 paper, with more detail in the associated electronic supplementary material [4]. We first considered the behaviour of breeding females, classifying species as social breeders if, during the course of the breeding season, several females that breed regularly share a common range, tolerate each other and form coherent groups—and as solitary breeders if they do not do so. This definition is intended to distinguish between species where females are solitary, accompany dependent young or aggregate outside the breeding season or only at foraging grounds and the relatively small number of mammals where social groups include multiple breeding females which share all areas of their range.

We classify species as socially monogamous if breeding females do not aggregate or share a common range and there is evidence that individual males and individual females form breeding pairs that persist for more than a single breeding season (in species where individuals reach maturity quickly, multiple breeding seasons might occur within a single year, though in most species pairs persist for more than 1 year). Here, our definition is intended to distinguish between the relatively large number of polygynous mammals where individual males guard single receptive females for minutes, hours or days before moving on to search for other partners and species where the sexes form bonded breeding pairs that persist for more than a single breeding season. Most monogamous mammals are iteroparous and we have not yet found firm evidence of any mammal which forms mixed sex pairs that share a common range and last throughout a breeding attempt but are not maintained across breeding attempts within years or across seasons (as is the case in many birds). However, two of the shrews listed in table 1 (*Cro. leucodon* and *Su. varilla*) may possibly do so and firm evidence of this would cause us to consider modifying our definition to include them and any other species where mixed sex pairs persist across individual breeding attempts but not across seasons.

Finally, we classify species as cooperative breeders where breeding adults are assisted in rearing young by non-breeders of either or both sexes. This is intended to distinguish between species where several breeding females share care of their offspring and are likely to gain direct fitness benefits from doing so or where non-breeding group members contribute to activities likely to generate mutualistic benefits (such as nest maintenance and predator defence) from species where non-breeding subordinates engage in activities that are unlikely to increase their own direct fitness, like feeding or carrying young born to other group members.

**Table 1.** Differences in the classification of shrews.

species	Do breeding females aggregate in groups?	Are aggregrations during the breeding season stable and cohesive?	Are individuals seen in mixed sex pairs during the breeding season?	Do males remain with the same female throughout the breeding season?	Do pairs remain stable across breeding seasons?	classification Lukas & Clutton-Brock	classification Valomy *et al*.
*Cryptotis parva*	no: aggregations dissolve and females build territories [14]	no	no	no	no	solitary	group
*Sorex cinereus*	no: aggregations appear to be of males chasing females [15]	no	no	no	no	solitary	group
*Sorex ornatus*	yes	no: frequent changes [16], and females intolerant [14]	no	no	no	solitary	group
*Crocidura leucodon*	no	no	yes	potentially	no: in autumn, shift to a gregarious way of life [17]	solitary	pair
*Crocidura russula*	no	no	uncertain: pairs occur, but high frequency of polygyny [18]	uncertain	no: during winter, shift to mixed sex aggregations [19]	solitary	pair
*Sorex coronatus*	no	no	yes	no: male home-ranges change during breeding season [20]	no: in autumn, males separate from females [20]	solitary	pair
*Suncus varilla*	no	no	yes	possibly, but there is only a single breeding attempt per year [21]	uncertain: partners appear to separate after breeding season [21]	solitary	pair

A further four species that we have classified as solitary breeders are classified as pair-living by Valomy *et al*. Contrasts in definitions are involved here, too. We classify species as socially monogamous (or pair-living) if there is evidence that they form stable mixed sex pairs which persist across breeding attempts or breeding seasons (the rationale for this is described in box 1 and is related to the need to distinguish between pair-living species and the large number of mammals where individual males guard single females for short periods during the breeding season before moving on to search for other partners) and regard socially monogamous species as a subset of species where breeding females do not aggregate with each other. Although the four species that Valomy *et al.* list as pair-living (*Crocidura leucodon*, *Crocidura russula*, *Sorex coronatus* and *Suncus varilla*) are commonly seen in mixed sex pairs during the breeding season, none of the studies they cite tracked the movements of substantive samples of identifiable individuals over time and it is not clear whether the same individuals associate with each other either throughout particular breeding attempts or across breeding attempts in the same season—and some observations suggest that they break up at the end of each breeding season. Moreover, all four species are short-lived and few pairs, if any, can persist across years. In this respect, shrews differ from some murid rodents, including species of *Microtus* and *Peromyscus*, where there is firm evidence that individuals form lifelong pairs which persist across breeding attempts and seasons (when new individuals start to reproduce) if both partners survive (e.g. [24–26]). As there is no firm evidence that individuals form pairs that persist across breeding attempts or seasons or that breeding females aggregate during the breeding season in these four shrew species, we classified these species as solitary breeders, too (table 1). A final difference is that we classified one species, *Suncus etruscus*, as monogamous because it was among the species listed in an early review by Kleiman & Malcolm [27] as being monogamous. However, as Schradin points out, this information is not sufficient to classify its social system and we now regard this as an error, as there is no evidence that pairs remain together across breeding attempts or seasons and we have found new reports that females are solitary and aggressive towards males outside the breeding season [28] and shall change its categorization in any future analysis. To check whether contrasts in the classifications of shrews between Valomy *et al.* and ourselves affected the conclusions of our analysis, we have re-run the comparisons included in our 2017 paper using Valomy *et al*.'s classifications and found that they do not (see electronic supplementary material, appendix S3). In retrospect, it would have been useful to have included a more detailed description and discussion of our classification of these eight species in our 2017 paper and to have explained the reasons why we did not follow Valomy *et al*.'s classifications.

Schradin's final criticism is that our 2017 paper draws on our 2013 dataset where we list all shrew species as solitary breeders. This is in line with most reviews of the social organization of shrews, which describe them as solitary breeders (see above); with the absence of evidence that breeding females aggregate in any of the 68 species for which we were able to find published information; and with the absence of group breeding (on our definition) in any other small, terrestrial insectivores or carnivores in our dataset. The principal comparisons in our 2017 paper that Schradin is commenting on did not include species whose social organization we inferred, as we compared the habitats of socially monogamous species and cooperative breeders (where all categorizations were based on published information). However, the paper contains a subsidiary comparison between the habitats occupied by cooperative breeders and those occupied by all other mammals, including 51 shrews for which we could find published information and 87 whose social organization we inferred to be similar (see electronic supplementary material, appendix S2). Here, we included data for the second group of species because the consensus view is that all shrews are solitary breeders and their inclusion helped to compensate for the under-representation of shrews as a result of the relative scarcity of studies. We agree with Schradin that there is always a danger that inclusion of species whose social organization is inferred may introduce error—though their exclusion has disadvantages, too (see below). A sensible course in situations of this kind would seem to be to repeat analyses with and without inferred data and we have consequently checked whether the inclusion of the shrews listed in our dataset for which no published reports were available could have affected the results of our comparisons. A re-run of our analyses shows that our results are unchanged if they are excluded (see electronic supplementary material, appendix S3). We also re-ran the comparisons using only Valomy *et al*.'s categorizations and found that, here too, our results are unaffected by these differences (see electronic supplementary material, appendix S3). As a case could be made that all shrew species should be omitted from comparative studies of the distribution of contrasting forms of social organization until their social behaviour has been studied in more detail, we also investigated whether the removal of all shrews from our comparisons affected their outcome. Here, too, we found that the outcome of the analysis was unchanged (see electronic supplementary material, appendix S3). We also checked the results of previous analyses that have included these categorizations and found that they, too, are unaffected by whether or not these species are included.

## 2. Broader issues

Schradin's commentary on our paper raises three general methodological issues regarding comparisons of major taxonomic groups based on categorizations of large samples of species. First, it emphasizes the importance of clear definitions and the need to be aware that contrasting definitions are often likely to cause the same species to be classified in different ways (table 1). Where the aim is to explore the distribution of traits across major taxonomic groups, the same definitions have to be used to classify very different species. This may require categories to be more specific and more restrictive in order to be applicable to diverse species [29,30], so definitions may diverge from those that might be used to compare more closely related species. Categories and definitions also need to be adjusted to the taxa involved and to the questions that are being asked—so they often vary between analyses of different taxa. For example, in our 2017 paper, we were principally interested in comparing the distribution of cooperative breeders with that of monogamous species where pairs persist for more than one season because phylogenetic reconstructions suggest that cooperative breeding has usually evolved in ancestors that formed long-lasting breeding pairs [3]. These issues are relevant to the use of published datasets by others: few datasets are constructed for general use and their purpose is usually to allow particular analyses to be checked and investigated. As a result, those that use published datasets for other analyses or add them to comparative databases they have compiled need to pay close attention to the definitions of categories that are being used in order to avoid combining information based on contrasting definitions. It is consequently important that comparative studies should provide clear and detailed descriptions of the definitions of their categories or variables.

A second point concerns the common problem that the distribution of information across species is often strongly affected by biases in the species that have been studied. Among mammals, the proportion of species that have been studied in any detail is much higher for diurnal, terrestrial and social species than it is for nocturnal, arboreal or solitary ones: for example, we have been able to locate published information on social behaviour for 62% of all diurnal mammals but only for 36% of all nocturnal species, including nocturnal shrews (see [31]). Data availability is also frequently geographically biased, with tropical and Southern Hemisphere species being less well represented than palaearctic ones [32,33]. As a result, comparisons of the incidence of female sociality across major taxonomic groups may need to compensate for biases in the distribution of species that have been studied. Where species from under-represented taxa, like shrews, show a high degree of conformity in their behaviour, a common approach has been to use the available information on the distribution of traits across taxa, combined with the number of species per taxon, to infer the traits of related species for which no direct data are available. In some cases, inference of this kind is based simply on the assumption that related species are likely to show the same traits, sometimes potentially supported by phylogenetic reconstructions—for example, a number of comparative studies of social behaviour (including two of the three studies that Schradin cites in his Commentary) have been based on datasets that assume that congeneric species share the same traits (e.g. [34–44]). In others, the categorization of species for which no direct data are available is inferred using ecological or life-history information in addition to phylogenetic proximity (for discussion, see [45]). At the end of his Commentary, Schradin argues against the use of inference and suggests that many comparisons may need to wait until enough detailed, high-quality datasets are available to allow quantitative comparisons to be made that do not rely on inference. We have much sympathy with his argument and, like him, believe that only long-term, individual-based studies provide reliable insights into the processes responsible for the diversity of social behaviour in mammals [46,47]. However, the proportion of species for which detailed, individual-based data are available from wild populations is likely to remain low for a long time—and we do not believe that it is sensible to delay asking questions about the distribution of major traits across different animal groups. As a result, we believe that, in some comparisons, it will be necessary to infer the traits of some species in order to reduce biases in representation. One sensible solution to this problem may be for analyses either to be repeated with and without inferred values to see whether results remained unchanged or to be repeated on one or more well-studied taxonomic groups where inference is unnecessary, such as primates or carnivores.

The final issue that Schradin's Commentary raises is the need for comparative studies to document the sources that they used to categorize species or to obtain data. Though some comparative studies have provided detailed information on their definitions and on their sources (e.g. [37,48,49]), the information associated with many comparative studies is not sufficient to allow specific values or categorizations to be linked to specific publications or understand whether values were inferred. The provision of more detailed and more extensive information is necessary to allow results to be checked and analyses to be explored (e.g. [30]) and journals that publish comparative studies need to be persuaded to relax constraints on the number of references that can be included or to facilitate publication of data and associated references elsewhere.

## Supplementary Material

Appendix 1

## Supplementary Material

Appendix 2: Information on shrew species

## Supplementary Material

Appendix 3: Results of comparative analyses

## Data Availability

Data are available in electronic supplementary material, appendix S2 as part of the electronic supplementary material (a table listing all shrew species with observed or inferred social system included in our 2017 analyses and additional species with observed social system included in our 2013 paper).
