# Supplementary material for: Comparative studies need to rely both on sound natural history data and on excellent statistical analysis
Source: R Soc Open Sci. 2017 Nov 15;4(11):171211. doi: 10.1098/rsos.171211 (PMC5717680; doi:10.1098/rsos.171211)
Supplement: Appendix 1 [file rsos171211supp1.docx]

**Appendix 1 to Lukas & Clutton-Brock Reply To Schradin (RSOS 2017): references used to classify shrews in our 2013 and 2017 papers.**

Balakrishnan, M. (1975). *Studies on mammalian behaviour aspects of ethology of the Indian musk shrew suncus murinus viridescens*. PhD Thesis. University of Kerala, Trivandrum.

Balloux, F., Goudet, J. & Perrin, N. (1998). Breeding system and genetic variance in the monogamous, semi‐social shrew, Crocidura russula. *Evolution*, 52(4), pp.1230-1235.

Bouteiller, C. & Perrin, N. (2000). Individual reproductive success and effective population size in the greater white–toothed shrew Crocidura russula. *Proceedings of the Royal Society of London B: Biological Science*s, 267(1444), pp.701-705.

Buckner C.H. (1969). Some aspects of the population ecology of the common shrew, Sorex araneus, near Oxford, England. *Journal of Mammalogy* 50, pp.326-332.

Burnie, D (editor). (2011). *Animal*. London, Dorling Kindersley.

Cantoni, D. & Vogel, P. (1989). Social organization and mating system of free- ranging, greater white-toothed shrews, Crocidura russula. *Animal Behaviour*, 38(2), pp.205-214.

Cantoni, D., & Rivier, L. (1992). Analysis of the secretions from the flank glands of 3 shrew species and their possible functions in a social context. In *Chemical Signals in Vertebrates 6 (eds. Doty & Mueller-Schwarze)* (pp.99-106). New York, Springer.

Cantoni, D. (1993). Social and spatial organization of free-ranging shrews, Sorex coronatus and Neomys fodiens (Insectivora, Mammalia). *Animal Behaviour*, 45(5), pp.975-995.

Choate, J. R. (1973). Cryptotys mexicana. *Mammalian Species* 28: pp.1-3.

Choate, J. R., Jones, J. K., Jones, C. (1994). *Handbook of Mammals of the South-Central States*. London, Louisiana State University Press.

Churchfield, J.S. (1979). *Studies on the ecology & behaviour of British shrews*. PhD Thesis. Queen Mary University, London.

Churchfield, S. (1984). Dietary separation in three species of shrew inhabiting water‐cress beds. *Journal of Zoology*, 204(2), pp.211-228.

Churchfield, S. (1990). *The natural history of shrews*. Cornell, Cornell University Press.

Churchfield, S., Hollier, J. & Brown, V.K. (1995). Population dynamics and survivorship patterns in the common shrew Sorex araneus in southern England. *Acta Theriologica*, 40(1), pp.53-68.

Crowcroft, P. (1955). Notes on the behaviour of shrews. *Behaviour*, 8(1), pp.63-80.

Dutton, J. & Haft, J. (1996). Distribution, ecology and status of an endemic shrew, Crocidura thomensis, from Sao Tomé. *Oryx*, 30(3), pp.195-201.

French, T.W. (1980). Natural history of the southeastern shrew, Sorex longirostris Bachman. *The American Midland Naturalist*, 104(1), pp.13-31.

George, S.B., Choate, J.R. & Genoways, H.H. (1986). Blarina brevicauda. *Mammalian Species*, (261), pp.1-9.

Getz, L.L. & McGuire, B. (2008). Factors influencing movement distances and home ranges of the short-tailed shrew (Blarina brevicauda). *Northeastern Naturalist*, 15(2), pp.293-302.

Getz, L.L. (1961). Factors influencing the local distribution of shrews. *The American Midland Naturalist*, 65(1), pp.67-88.

Haberl, W. (1996). Fostering in European common shrews Sorex araneus (Soricidae, Insectivora). *Acta Theriologica*, 41(4), pp.433-438.

Haltenorth, T. & Diller, H. (1980). *A field guide to the mammals of Africa including Madagascar*. London: Collins.

Hawes, M.L. (1977). Home range, territoriality and ecological separation in sympatric shrews, Sorex vagrans and Sorex obscurus. *Journal of Mammalogy*, 58(3), pp.354-367.

Hays, W.S. & Lidicker, W.J., (2000). Winter aggregations, Dehnel effect, and habitat relations in the Suisun shrew Sorex ornatus sinuosus. *Acta Theriologica*, 45(4), pp.433-442.

Ichikawa, A., Nakamura, H., & Yoshida, T. (2005). Mark-recapture analysis of the Japanese water shrew Chimarrogale platycephala in the Fujisawa Stream, a tributary of the Tenryu River, central Japan. *Mammal Study*, *30*(2), pp.139-143.

Inoue T. (1991). Sex differences in spatial distribution of the big-clawed shrew Sorex unguiculatus. *Acta Theriologica*, 36(3-4), pp.229-237.

Jameson Jr, E.W. (1955). Observations on the biology of Sorex trowbridgei in the Sierra Nevada, California. *Journal of Mammalogy*, 36(3), pp.339-345.

Kirkland, G. L., Merritt, J. F., & Rose, R. K. (Eds.). (1994). *Advances in the biology of shrews*. Pittsburgh, Carnegie Museum of Natural History.

Kirkland, G.L. & Schmidt, D.F. (1996). Sorex arcticus. *Mammalian Species*, (524), pp.1-5.

Krushinska, N.L. and Rychlik, L. (1993). Intra-and interspecific antagonistic behaviour in two sympatric species of water shrews: Neomys fodiens and N. anomalus. *Journal of Ethology*, 11(1), pp.11-21.

Krushinska, N.L. & Rychlik, L. (1994). Aggressiveness of a Neomys fodiens parous female towards conspecific and N. anomalus intruders. *Acta Theriologica*, 39(3), pp.329-332.

Krushinska, N.L., Rychlik, L. & Pucek, Z. (1994). Agonistic interactions between resident and immigrant sympatric water shrews: Neomys fodiens and N. anomalus. *Acta Theriologica*, 39(3), pp.227-247.

Lin, T.T., You, E.M. & Lin, Y.K. (2009). Social and genetic mating systems of the Asian lesser white-toothed shrew, Crocidura shantungensis, in Taiwan. *Journal of Mammalogy*, 90(6), pp.1370-1380.

Lord Medway. (1969). *The wild mammals of Malaya (Peninsular Malaysia) and Singapore*. New York, Oxford University Press.

Lynch, C.D. (1991). Population dynamics in the Lesser dwarf shrew, Suncus varilla (Mammalia: Soricidae): results. *Navorsinge van die Nasionale Museum: Researches of the National Museum*, 7(9), pp.467-470.

Magnanou, E., Attia, J., Fons, R., Boeuf, G. & Falcon, J. (2009). The timing of the shrew: continuous melatonin treatment maintains youthful rhythmic activity in aging Crocidura russula. *PloS one*, 4(6), p.e5904.

Maier, T.J. & Doyle, K.L. (2006). Aggregations of masked shrews (Sorex cinereus): density-related mating behavior?/Agrégation de musaraignes masquées (Sorex cinereus): accouplement relatif à la densité de la population?. *Mammalia*, 70(1-2), pp.86-89.

Massachusetts Division of Fisheries & Wildlife. (2012). *Sorex palustris*. http://masslib-dspace.longsight.com/bitstream/handle/2452/423148/ocn954244907.pdf

McCay, T.S. (2001). Blarina carolinensis. *Mammalian Species*, pp.1-7.

McDevitt, R.M. & Andrews, J.F. (1994). The importance of nest utilization as a behavioural thermoregulatory strategy in Sorex minutus the pygmy shrew. *Journal of Thermal Biology,* 19(2), pp.97-102.

Merritt, J.F. (1986). Winter survival adaptations of the short-tailed shrew (Blarina brevicauda) in an Appalachian montane forest. *Journal of Mammalogy*, 67(3), pp.450-464.

Murariu, D. & Benedek, A.M. (2005). New reports on the presence of Sorex alpinus Schinz, 1837 (Insectivora: Soricidae) in the southern Carpathians (Romania). *Travaux du Muséum National d'Histoire Naturelle ‘Grigore Antipa*, 48, pp.395-405.

Nagorsen, D.W. (1996). *Opossums, shrews and moles of British Columbia* (Vol. 2). Vancouver, University of British Columbia Press.

Nowak, R.M. (1999). *Walker's mammals of the world*. London, The John Hopkins University Press.

Ochocińska, D. & Taylor, J.R. (2005). Living at the physiological limits: field and maximum metabolic rates of the common shrew (Sorex araneus). *Physiological and Biochemical Zoology*, 78(5), pp.808-818.

Ohdachi, S. (1992). Home ranges of sympatric soricine shrews in Hokkaido, Japan. *Acta Theriologica*, 37(1-2), pp.91-101.

Platt, W.J. (1976). The social organization and territoriality of short-tailed shrew (Blarina brevicauda) populations in old-field habitats. *Animal Behaviour*, 24(2), pp.305-318.

Rychlik, L. & Zwolak, R. (2005). Behavioural mechanisms of conflict avoidance among shrews. *Acta theriologica*, 50(3), pp.289-308.

Rychlik, L. & Zwolak, R. (2006). Interspecific aggression and behavioural dominance among four sympatric species of shrews. *Canadian Journal of Zoology*, 84(3), pp.434-448.

Rychlik, L. (1998). Evolution of social systems in shrews. In: *Evolution of shrews* (eds. Wojcik & Wolsan) Białowieza, Poland: Mammal Research Institute, Polish Academy of Sciences. pp. 347-406.

Rychlik, L., Ruczyński, I. & Borowski, Z. (2010). Radiotelemetry Applied to Field Studies of Shrews. *Journal of Wildlife Management*, 74(6), pp.1335-1342.

Sanborn, C.C. & Hoogstraal, H. (1953). Some Mammals of Yemen and their Ectoparasites. *Fieldiana: Zoology*, 34(23), pp.229-52.

Shchipanov, N.A., Kalinin, A.A., Demidova, T.B., Oleinichenko, V.Y., Aleksandrov, D.Y., Kouptzov, A.V., Merritt, J.F., Churchfield, S., Hutterer, R. & Sheftel, B.I. (2005). Population ecology of red-toothed shrews, Sorex araneus, S. caecutiens, S. minutus, and S. isodon, in central Russia. In: *Kirkland et al.,* pp.201-216.

Shen, Y. W., Lia, W. B., & Hu, J. C. (2005). Study on the Spatial Distribution Patterns of Anouroserex squamipes in Nanchong, Sichuan Province. *Journal of Xihua Teachers College (Natural Science)*, *2*, 008.

Shortridge, G.C. (1934). *The mammals of south west Africa*. London: Heinemann.

Simeonovska-Nikolova, D. M. (2004). Seasonal changes in social behaviour and spatial structure of Crocidura leucodon in north-western Bulgaria. *Acta theriologica*, *49*(2), 167-179.

Skinner, J.D. & Chimimba, C.T. (2005). *The mammals of the southern African sub-region*. Cambridge, Cambridge University Press.

Smithers, R.H., 1983*. The mammals of the southern African subregion*. Pretoria, University of Pretoria Press.

Stockley P., Searle J.B., Macdonald D.W. & Jones C.S. (1994). Alternative reproductive tactics in male common shrews: relationships between mate-searching behavior, sperm production, and reproductive success as revealed by DNA fingerprinting. *Behavioural Ecology and Sociobiology* 34(1), 71-78

Stockley, P., Searle, J.B., Macdonald, D.W. & Jones, C.S. (1993). Female multiple mating behaviour in the common shrew as a strategy to reduce inbreeding. *Proceedings of the Royal Society of London B: Biological Sciences*, 254(1341), pp.173-179.

Stuart, C., & Stuart, T. (2001). *Field guide to mammals of southern Africa*. Cape Town, Struik.

Thompson, C.W., Choate, J.R., Genoways, H.H. & Finck, E.J. (2011). Blarina hylophaga (Soricomorpha: Soricidae). *Mammalian Species*, 43(1), pp.94-103.

Volodin, I.A., Zaytseva, A.S., Ilchenko, O.G., Volodina, E.V. & Chebotareva, A.L. (2012). Measuring airborne components of seismic body vibrations in a Middle- Asian sand-dwelling Insectivora species, the piebald shrew (Diplomesodon pulchellum). *Journal of Experimental Biology*, 215(16), pp.2849-2852.

Vololomboahangy, R. & Goodman, S. (2008). Suncus madagascariensis. *The IUCN Red List of Threatened Species*: e.T41439A10472305. http://dx.doi.org/10.2305/IUCN.UK.2008.RLTS.T41439A10472305.en

Yu, H.T., Cheng, T.W. & Chou, W.H. (2001). Seasonal activity and reproduction of two syntopic white-toothed shrews (Crocidura attenuata and C. kurodai) from a subtropical montane forest in central Taiwan. *Zoological Studies Taipei*, 40(2), pp.163-169.

Zuri, I. & Rado, R. (2000). Sociality and agonistic behavior in the lesser white- toothed shrew, Crocidura suaveolens. *Journal of Mammalogy*, 81(2), pp.606-616.

*References published since the publication of the initial data in 2013:*

Kingdon, J., Happold, D. C. D., Butynski, T. M., Hoffmann, M., Happold, M., & Kalina, J. (2013). *Mammals of Africa*. London, Bloomsbury.

Klenovšek, T., Novak, T., Čas, M., Trilar, T., & Janžekovič, F. (2013). Feeding ecology of three sympatric Sorex shrew species in montane forests of Slovenia. *Folia Zool*, *62*(3), 193-199.

Merritt, J.F. & Zegers, D.A. (2014). Social thermoregulation in least shrews, Cryptotis parva. *Mammalia*, 78(1), pp.11-22.
