# Supplementary material for: Comparative studies need to rely both on sound natural history data and on excellent statistical analysis
Source: R Soc Open Sci. 2017 Nov 15;4(11):171211. doi: 10.1098/rsos.171211 (PMC5717680; doi:10.1098/rsos.171211)
Supplement: Appendix 2: Information on shrew species [file rsos171211supp2.docx]

**Appendix 2 to Lukas & Clutton-Brock Reply To Schradin (RSOS 2017):**

List of shrew species with observed or inferred social system included in our 2017 analyses and additional species with observed social system included in our 2013 paper. Full references are provided in Appendix 1.

| **Shrew species included in 2017** | **Social System Inferred?** | **Reference** |
| --- | --- | --- |
| Anourosorex_squamipes | No | Kirkland et al. 1994; Shen et al. 2005 |
| Blarina_brevicauda | No | Platt 1976; George et al. 1986; Merritt 1986; Getz & McGuire 2008 |
| Blarina_carolinensis | No | McCay 2001 |
| Blarina_hylophaga | No | Thompson et al. 2011 |
| Blarinella_quadraticauda | Yes |  |
| Chimarrogale_hantu | Yes |  |
| Chimarrogale_himalayica | Yes |  |
| Chimarrogale_phaeura | Yes |  |
| Chimarrogale_platycephalus | No | Ichikawa et al. 2005 |
| Chimarrogale_styani | Yes |  |
| Chodsigoa_parca | Yes |  |
| Chodsigoa_smithii | Yes |  |
| Crocidura_batesi | Yes |  |
| Crocidura_beatus | Yes |  |
| Crocidura_bottegi | Yes |  |
| Crocidura_crossei | Yes |  |
| Crocidura_cyanea | Yes |  |
| Crocidura_dolichura | Yes |  |
| Crocidura_douceti | Yes |  |
| Crocidura_elgonius | Yes |  |
| Crocidura_flavescens | No | Shortridge 1934, Kingdon et al. 2013 |
| Crocidura_foxi | Yes |  |
| Crocidura_fuliginosa | No | Medway 1969 |
| Crocidura_fulvastra | Yes |  |
| Crocidura_fumosa | Yes |  |
| Crocidura_fuscomurina | No | Rychlik 1998; Stuart & Stuart 2001 |
| Crocidura_grandiceps | Yes |  |
| Crocidura_grassei | Yes |  |
| Crocidura_grayi | Yes |  |
| Crocidura_hildegardeae | Yes |  |
| Crocidura_hirta | No | Smithers 1983; Rychlik 1998 |
| Crocidura_horsfieldii | No | Haltenorth & Diller 1980 |
| Crocidura_jacksoni | Yes |  |
| Crocidura_jouvenetae | Yes |  |
| Crocidura_leucodon | No | Rychlik 1998; Rychlik & Zwolak 2005 |
| Crocidura_littoralis | Yes |  |
| Crocidura_ludia | Yes |  |
| Crocidura_luna | Yes |  |
| Crocidura_lusitania | Yes |  |
| Crocidura_maquassiensis | Yes |  |
| Crocidura_mariquensis | No | Smithers 1983; Rychlik 1998 |
| Crocidura_maurisca | Yes |  |
| Crocidura_monax | Yes |  |
| Crocidura_montis | Yes |  |
| Crocidura_mutesae | Yes |  |
| Crocidura_nanilla | Yes |  |
| Crocidura_nigeriae | Yes |  |
| Crocidura_nigricans | Yes |  |
| Crocidura_nigrofusca | Yes |  |
| Crocidura_niobe | Yes |  |
| Crocidura_olivieri | No | Rychlik 1998; Stuart & Stuart 2001 |
| Crocidura_pasha | Yes |  |
| Crocidura_planiceps | Yes |  |
| Crocidura_poensis | No | Kirkland et al. 1994 |
| Crocidura_religiosa | Yes |  |
| Crocidura_russula | No | Cantoni & Vogel 1989; Balloux et al. 1998; Bouteiller & Perrin 2000; Magnanou et al. 2009 |
| Crocidura_silacea | Yes |  |
| Crocidura_suaveolens | No | Zuri & Rado 2000 |
| Crocidura_tarfayensis | Yes |  |
| Crocidura_theresae | Yes |  |
| Crocidura_turba | Yes |  |
| Crocidura_usambarae | Yes |  |
| Crocidura_viaria | No | Kirkland et al. 1994 |
| Crocidura_whitakeri | Yes |  |
| Crocidura_yankariensis | Yes |  |
| Cryptotis_goldmani | Yes |  |
| Cryptotis_goodwini | Yes |  |
| Cryptotis_magna | Yes |  |
| Cryptotis_meridensis | Yes |  |
| Cryptotis_mexicana | No | Choate 1973 |
| Cryptotis_montivaga | Yes |  |
| Cryptotis_nigrescens | Yes |  |
| Cryptotis_parva | No | Getz 1961, Merritt & Zegers 2014 |
| Cryptotis_squamipes | Yes |  |
| Cryptotis_thomasi | Yes |  |
| Diplomesodon_pulchellum | No | Volodin et al. 2012 |
| Feroculus_feroculus | Yes |  |
| Megasorex_gigas | Yes |  |
| Myosorex_cafer | No | Smithers 1983; Rychlik 1998 |
| Myosorex_varius | No | Smithers 1983; Rychlik 1998 |
| Nectogale_elegans | No | Kirkland et al. 1994 |
| Neomys_anomalus | No | Krushinka et al. 1994; Rychlik & Zwolak 2005; 2006 |
| Neomys_fodiens | No | Krushinka et al. 1994; Rychlik & Zwolak 2005; 2006 |
| Neomys_teres | Yes |  |
| Notiosorex_crawfordi | No | Kirkland et al. 1994; Rychlik 1998 |
| Notiosorex_evotis | Yes |  |
| Paracrocidura_schoutedeni | Yes |  |
| Ruwenzorisorex_suncoides | Yes |  |
| Scutisorex_somereni | No | Burnie 2011 |
| Sorex_alpinus | No | Klenovsec et al. 2013 |
| Sorex_arcticus | No | Kirkland & Schmidt 1996 |
| Sorex_asper | Yes |  |
| Sorex_bairdi | Yes |  |
| Sorex_bendirii | No | Nagorsen 1996 |
| Sorex_caecutiens | No | Kirkland et al. 1994, Shchipanov et al. 2005 |
| Sorex_cinereus | No | Kirkland et al. 1994, Maier & Doyle 2006 |
| Sorex_coronatus | No | Cantoni & Rivier 1992; Cantoni 1993 |
| Sorex_daphaenodon | No | Kirkland et al. 1994 |
| Sorex_dispar | Yes |  |
| Sorex_fumeus | No | Kirkland et al. 1994; Rychlik 1998 |
| Sorex_granarius | Yes |  |
| Sorex_hosonoi | Yes |  |
| Sorex_hoyi | No | Kirkland et al. 1994 |
| Sorex_isodon | No | Kirkland et al. 1994, Shchipanov et al. 2005 |
| Sorex_jacksoni | Yes |  |
| Sorex_longirostris | No | French 1980; Rychlik 1998 |
| Sorex_minutissimus | No | Kirkland et al. 1994; Rychlik 1998 |
| Sorex_minutus | No | Churchfield 1984; McDevitt & Andrews 1994; Shchipanov et al. 2005 |
| Sorex_mirabilis | Yes |  |
| Sorex_monticolus | No | Kirkland et al. 1994; Rychlik 1998 |
| Sorex_nanus | Yes |  |
| Sorex_ornatus | No | Kirkland et al. 1994; Rychlik 1998; Hays & Lidicker 2000 |
| Sorex_pacificus | Yes |  |
| Sorex_palustris | No | Rychlik 1998; Massachusetts Division of Fisheries & Wildlife 2012 |
| Sorex_preblei | Yes |  |
| Sorex_roboratus | No | Kirkland et al. 1994 |
| Sorex_samniticus | Yes |  |
| Sorex_tenellus | Yes |  |
| Sorex_tundrensis | No | Kirkland et al. 1994; Rychlik 1998 |
| Sorex_unguiculatus | No | Kirkland et al. 1994; Inoue 1991; Ohdachi 1992 |
| Sorex_vagrans | No | Hawes 1977; Rychlik 1998 |
| Soriculus_nigrescens | Yes |  |
| Suncus_etruscus | Now: Solitary | Kingdon et al. 2013 |
| Suncus_infinitesimus | Yes |  |
| Suncus_lixus | Yes |  |
| Suncus_madagascariensis | No | Vololomboahangy & Goodman 2008 |
| Suncus_malayanus | Yes |  |
| Suncus_megalura | Yes |  |
| Suncus_murinus | No | Sanborn & Hoogstral 1953; Balakrishnan 1975 |
| Suncus_remyi | Yes |  |
| Suncus_varilla | No | Lynch 1991 |
| Surdisorex_norae | Yes |  |
| Surdisorex_polulus | Yes |  |
| Sylvisorex_johnstoni | Yes |  |
| Sylvisorex_lunaris | Yes |  |
| Sylvisorex_ollula | Yes |  |
| Sylvisorex_vulcanorum | Yes |  |
|  |  |  |
| **Shrew species included in 2013** | **Social System Inferred?** | **Reference** |
| Crocidura_attenuata | No | Yu et al. 2001 |
| Crocidura_bicolor | No | Shortridge 1934 |
| Crocidura_deserti | No | Shortridge 1934 |
| Crocidura_kurodai | No | Yu et al. 2001 |
| Crocidura_nyansae | No | Shortridge 1934 |
| Crocidura_shortridgei | No | Shortridge 1934 |
| Crocidura_thomensis | No | Dutton & Haft 1996 |
| Crocidura_ shantungensis | No | Lin et al. 2009 |
| Sorex_araneus | No | Buckner 1969; Stockley et al. 1993; Churchfield et al. 1995; Ochocinska et al. 2005 |
| Sorex_gracillimus | No | Ohdachi 1992 |
| Sorex_obscurus | No | Hawes 1977 |
| Sorex_trowbridgei | No | Jameson 1955 |
| Suncus_caeruleus | No | Haltenorth & Diller 1980 |
| Suncus_giganteus | No | Haltenorth & Diller 1980 |
| Suncus_infinitesimes | No | Smithers 1983 |
| Suncus_montanus | No | Haltenorth & Diller 1980 |
| Sylvisorex_megalura | No | Smithers 1983 |
