# Supplementary material for: Comparative studies need to rely both on sound natural history data and on excellent statistical analysis
Source: R Soc Open Sci. 2017 Nov 15;4(11):171211. doi: 10.1098/rsos.171211 (PMC5717680; doi:10.1098/rsos.171211)
Supplement: Appendix 3: Results of comparative analyses [file rsos171211supp3.docx]

**Appendix 3 to Lukas & Clutton-Brock Reply To Schradin (RSOS 2017): results of repeated analyses with different subsets of shrew data.**

*Original comparison of rainfall in the habitats of cooperative breeders vs all other mammals, as reported in our 2017 paper:*

Predictors of distribution of cooperative breeding across mammals:

social monogamy post-mean 43.12 [95%CI 18.83 – 61.14], p<0.001;

annual rainfall post-mean -0.05 [95%CI -0.13– -0.01], p=0.003;

sample size: 1351 non- versus 32 cooperative breeders).

*Repeated comparison of rainfall in the habitats of cooperative breeders vs all other mammals, including shrews as classified by Valomy et al (2015):*

Predictors of distribution of cooperative breeding across mammals:

social monogamy post-mean 28.57 [95%CI 13.76 – 41.32], p<0.001;

annual rainfall post-mean -0.05 [95%CI -0.09 – -0.02], p<0.001;

sample size: 1222 non- versus 32 cooperative breeders).

*Repeated comparison of rainfall in the habitats of cooperative breeders vs all other mammals, removing all shrew species with no primary information:*

Predictors of distribution of cooperative breeding across mammals:

social monogamy post-mean 15.98 [95%CI 8.46 – 27.18], p<0.001;

annual rainfall post-mean -0.03 [95%CI -0.04 – -0.01], p=0.005;

sample size: 1262 non- versus 32 cooperative breeders).

*Repeated comparison of rainfall in the habitats of cooperative breeders vs all other mammals, removing all shrew species:*

Predictors of distribution of cooperative breeding across mammals:

social monogamy post-mean 24.50 [95%CI 17.99 – 31.81], p<0.001;

annual rainfall post-mean -0.06 [95%CI -0.10 – -0.02], p=0.001;

sample size: 1213 non- versus 32 cooperative breeders).
